# Low Number of Owner-Reported Suspected Transmission of Foodborne Pathogens From Raw Meat-Based Diets Fed to Dogs and/or Cats

**DOI:** 10.3389/fvets.2021.741575

**Published:** 2021-10-12

**Authors:** Nicole Renee Cammack, Ryan Michael Yamka, Vicki Jean Adams

**Affiliations:** ^1^Independent Researcher, Watertown, CT, United States; ^2^Independent Researcher, Luna Science & Nutrition, Trumbull, CT, United States; ^3^Veterinary Epidemiology Consultant, Vet Epi, Suffolk, United Kingdom

**Keywords:** commercial pet food, pathogens, food safety, dogs (*Canis lupus familiaris*), cats, minimally processed diets, raw meat-based diet, commercial pet food

## Abstract

The aim of this worldwide survey was to determine owner-reported frequency of pathogen transmission to humans living in or in contact with households feeding their pets raw, minimally processed (MP) diets. A total of 5,611 responses were gathered from 62 countries with 77.1% of households feeding only MP diets to dog and/or cat(s) with no confirmed cases of pathogen transmission or infection by laboratory testing. Eleven households (0.20%; 95% CI, 0.10–0.36) were classified as having experienced “probable” transmission, and 20 households (0.36%; 95% CI, 0.22–0.56) were classified as having experienced “possible” transmission to result in a total of 31 households (0.55%; 95% CI, 0.38–0.79) being identified as potential cases of transmission. The remainder of households (*n* = 5,580 = 99.45%; 95% CI, 99.21–99.62) were not considered to have experienced potential transmission of foodborne pathogens based on their responses to the survey. The most frequently reported pathogens were *Salmonella* (*n* = 11, 0.2%), *Campylobacter* (*n* = 6, 0.1%), and *Escherichia coli* (*n* = 4, 0.1%), with the most common age group being adults age 18–65 (*n* = 29, 78.4% of cases). Beef and chicken were the most common proteins reported as being fed in case households, although this was not associated with pathogen transmission. Households feeding a greater number of different protein sources, including pork, turkey, duck, rabbit, and salmon, were associated with decreased risk of pathogen transmission. Additional risk factors associated with pathogen transmission included preparing either MP diets in a separate location, with different utensils than human food, mixing MP diets with dry (kibble) diets and feeding a limited variety of protein sources. Based on the results of this survey, confirmed pathogen transmission from MP diets to humans appears to be rare. We conclude that potential or probable cases of pathogen transmission is likely dependent upon hygiene and food safety measures, and more education surrounding food safety should reduce risk.

## Introduction

The popularity of feeding minimally processed (MP), or raw, diets to companion dogs and cats has been increasing in popularity in recent years. Previously known as raw-meat-based diets (RMBDs), MP diets are raw (uncooked) meat-based diets and loosely defined as striated muscle, which is typically skeletal or found in the tongue, diaphragm, heart and/or esophagus with or without fat, tendons, and bones (1), and can be made commercially or at home. Other organs may also be utilized including lungs, liver, spleen, and kidney. These diets may or may not include the use of cooked or raw fruits and vegetables, raw dairy products, and raw poultry eggs. The practice of feeding of minimally processed diets can be grouped into two major types: MP commercial diets (MPCDs) or MP home-prepared diets (MPHDs) (2). MP diets of either type may be “complete and balanced” or unbalanced. Complete and balanced commercial products, according to the Association of American Feed Control Officials (AAFCO), are intended to be fed as the main source of nutrition for designated life stages, while unbalanced products are intended to be fed supplemental to kibble, canned, or other balanced diets.

Concerns regarding MP diets and their potential risk to human health arise from instances of raw meat products containing pathogens in the USA and around the world (3). It has been documented that canines and felines often shed raw-meat-associated pathogens in their feces, which does support cause for concern (4–7). There have been documented cases of people and pets becoming ill from MP, although documented cases appear to largely be from MPCD, as tracking and identifying cases from MPHD would be present challenges unless the source of contamination was the human food supply. For instance, between 2018 and 2019 the Centers for Disease Control (CDC) conducted an investigation into both human turkey products and MPCD with turkey containing *Salmonella*, which was also found in turkey for human consumption (8). Several other incidents of pathogen contamination of MPCD have resulted in recalls of which some have reports of ill humans and/or pets, and some do not (3, 9, 10), although each of these challenges is not exclusive to MP, since there are numerous documented cases of pathogen contamination of dry diets and treats as well (11–13). In comparison to livestock or wild animals, the role of dogs and cats as pathogen carriers is unique in that they can interact with urban, wilderness, and domestic environments, and each environment holds independent risk factors that are also independent of feeding practices or preferences (14). Ultimately, there are multiple variables involved in the potential for companion animals and humans to become infected with pathogens, and most of these variables have never been quantified.

Pathogens of interest for MP include species of *Salmonella, Escherichia coli, Campylobacter, Listeria*, among others. The American Animal Hospital Association (AAHA), American Veterinary Medical Association (AVMA), and other veterinary organizations discourage the practice of feeding MP due to pathogenic risks to both dogs, cats, and humans (15, 16). Some data used to support this claim are inadequate and do not relate to MP, since one reference is tied to *Salmonella* in pig ear treats (5, 17). It is not clear if the studies that the FDA and the AVMA cite involve MP commercial diets with established pathogen control (e.g., high pressure processing) or if they include both MPCD and MPHD. Both MPCD and MPHD have unique pathogenic risk factors because MPCDs are held to the same standard (e.g., zero tolerance policy) as kibble and other food formats, whereas MPHD, if made from meats from the human food supply, would be expected to harbor pathogens, as these meats are sold with the intent of being cooked.

Regardless of pathogen contamination of the diet, the true risk and prevalence of transmission from MP diets and/or pet to human is largely unknown. A study reported a total of 63 cases of owner reported transmission of one or more foodborne pathogens from 16,475 households responding to the survey; 39 of these cases were termed “confirmed transmission,” as human fecal samples were submitted to a laboratory for testing, and an additional 24 cases were termed “suspected transmission,” as there was no laboratory confirmation or testing (18). The risk and prevalence of humans becoming infected with pathogens from feeding MP diets or handling feces from dogs consuming MP diets or other diets has not been established despite documented contamination of all types of pet food formats.

The aim of this study was to estimate owner-reported frequency of transmission of pathogens to humans living in or in contact with households (HHs) that reported feeding MP diets on their own or in conjunction with another pet food form to their cats and/or dog. We also aimed to identify additional factors associated with the occurrence of reported pathogen transmission that were not related to diet.

## Materials and Methods

### Survey Design and Procedure

This survey used an online questionnaire to collect information on feeding practices and the potential prevalence of pathogenic transmission to humans among HH feeding MP as a whole diet or as a supplement to their cats or dogs. The survey questionnaire used was adapted from the survey used by Anturaniemi et al. (18). All of the original questions and two additional questions were used in the present study. The online survey link was open from January 5, 2020 and closed early on March 17, 2020 (73 days) due to the global coronavirus disease 2019 (COVID-19) pandemic and the potential for GI symptoms in humans related to the virus to bias the reporting of potential cases. The survey was targeted to owners who currently feed or have fed MP diets to their dog(s) and/or cat(s) and was distributed in English via invitation with a link to the online survey sent to a wide variety of personal and public social media accounts, social media-based MP diet feeding groups, veterinary practices, and individual email addresses. An effort was made to distribute this survey among both “pro-MP diet” and “anti-MP diet” groups and not to limit it to one or the other side of the debate.

### Potential Transmission of Foodborne Pathogens

Regression analysis was planned for responses from the survey. Potential cases of transmission of foodborne pathogens within a household were defined in two ways, similar to how they were classified in the study by Anturaniemi et al. (18). However, the terms “probable” and “possible” were used in place of “confirmed” and “suspected” for potential cases within a household due to the lack of submitted laboratory reports to confirm cases. HHs that responded “Yes” to both of the following questions were classified as “probable” cases of transmission:

#30—Are there/have there been PEOPLE in your household that have become sick from handling the raw pet food or that have become sick from contact with the raw food eating pet?
#31—Was this pathogenic transmission (i.e., bad bacteria, germs etc.) verified by a doctor via a stool (fecal) sample?

HHs that responded “yes” to one or the other of these two questions were classified as “possible” cases of transmission.

### Statistical Analysis

Data were downloaded from the online survey platform “Survey Monkey” as an Excel file (^*^.xlsx) and then imported into SPSS for statistical analysis. Frequencies are reported as number of responses (N) with percentages (%) and 95% confidence intervals (CI) where appropriate. Results of cross-tabulations with chi-square or Fisher's exact tests are reported with odds ratios (OR) and 95% CIs.

Logistic regression analysis was planned using a dichotomous outcome variable to define a case of transmission coded as 1 and a control coded as 0. Using the specified definitions of “probable” and “possible” gave rise to two datasets for logistic regression analysis: the first analysis included just the “probable” cases with a tighter definition, and the second analysis included both the “probable” and “possible” HH as cases (coded as 1). Both analyses used the HH with no suspected potential of transmission as controls (coded as 0). The potential independent predictor variables were created from the responses to other questions asked in the survey including country, type of pet fed raw, age groups of people living in the household, etc. (**Table 3**). Variables with *p* ≤ 0.25 were considered for inclusion in backwards regression modeling. Results of logistic regression are reported with ORs and 95% CIs. The level of significance for inclusion of variables in the final model was 0.05.

## Results

### Demographics

A total of 5,640 responses were gathered, which resulted in 5,611 useable responses. Only responses from completed questionnaires were used. The majority of respondents were female (>90%), and responses came from a total of 62 countries with 45.3% from the USA, 33.1% from the UK, 11.2% from Canada, and 3.6% from Australia. The remaining 6.8% of respondents were from 58 other countries (Table 1). Most HH reported only feeding raw food to a dog or dogs (77.1%, Table 2). The remaining respondents indicated feeding raw to both cats and dogs (16.6%, Table 2) and cats only (6.3%, Table 2). HH reported 69.8% being adults only (18+) and 30.2% with household members under 18 years of age. Of the HH, 15.2% reported having members above 65 years of age, and 17.1% reported at least one member of their household being immunocompromised. At least one child was attending daycare in 6.3% of HH.

**Table 1:** Countries responding to the survey with numbers (N) and percentages (%) of respondent households (HHs), “probable” and “possible” cases of transmission and HHs with no transmission reported.

			**Cases of transmission**					
	**Households responding**		**“Probable” cases**		**“Possible” cases**		**No transmission**	
**Country**	***N***	**%**	***N***	**%**	***N***	**%**	***N***	**%**
USA	2,543	45.32	6	0.11	10	0.18	2,527	45.04
UK	1,858	33.11	4	0.07	1	0.02	1,853	33.02
Canada	629	11.21	0	0.00	4	0.07	625	11.14
Australia	200	3.56	0	0.00	1	0.02	199	3.55
Other	381	6.79	1	0.02	4	0.07	376	6.70
**Total**	5,611	100	11	0.20	20	0.36	5,580	99.45

**Table 2:** Frequencies (*N* = number and % = percent) of types of pets reported living in the households responding to the survey.

**Type or Pet**	***N***	**%**
Dog or dogs only	4,327	77.1
Dog(s) and cat(s)	929	16.6
Cat or cats only	355	6.3
**Total**	5,611	100.0

### MP Diet Handling and Feeding Practices

**Figure 1:**
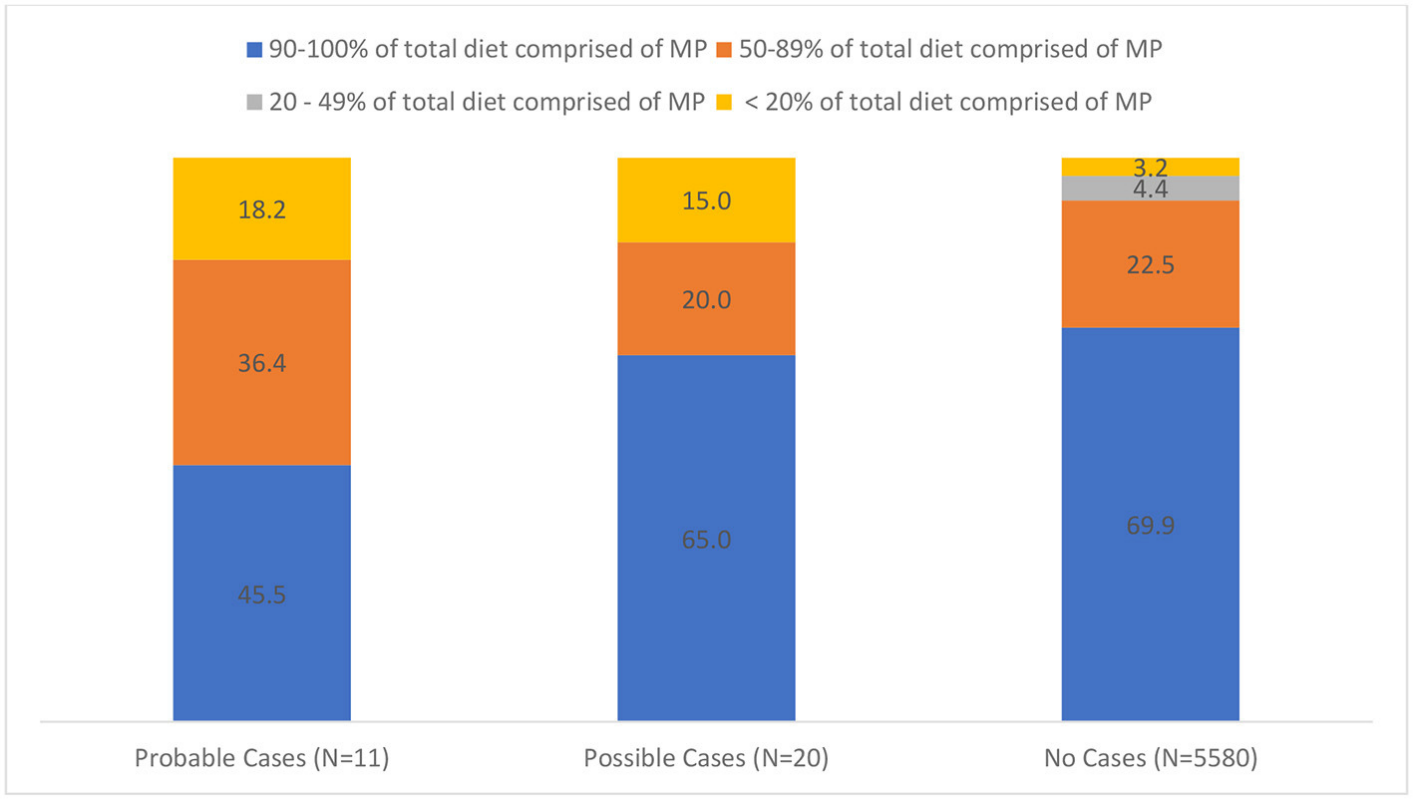
Distribution of probable, possible, and no cases of pathogens in households feeding various percentages of minimally processed diets to cats and/or dogs.

HHs were divided into two types: those reporting feeding MPHD (51.1%) and those that reported feeding MPCD (48.9%). HHs were also categorized into three categories depending on whether they reported MPHD only (19.9%), MPCD only (48.9%), and those that fed a combination of both (31.2%). Responses indicated that 69.8 of HH fed 90–100% MP diets, 22.5% fed 50–89% MP diets, 4.4% fed 20–49%, and 3.3% fed <20% MP diets. Respondents indicated the remaining diet consisted of kibble, canned, freeze dried, and cooked diets at 15.3, 9, 22, and 14.2%, respectively. MP diets that were not mixed with another food form (e.g., kibble) were demonstrated to have a lesser prevalence of pathogen transmission within this survey (Figure 1).

**Figure 2:**
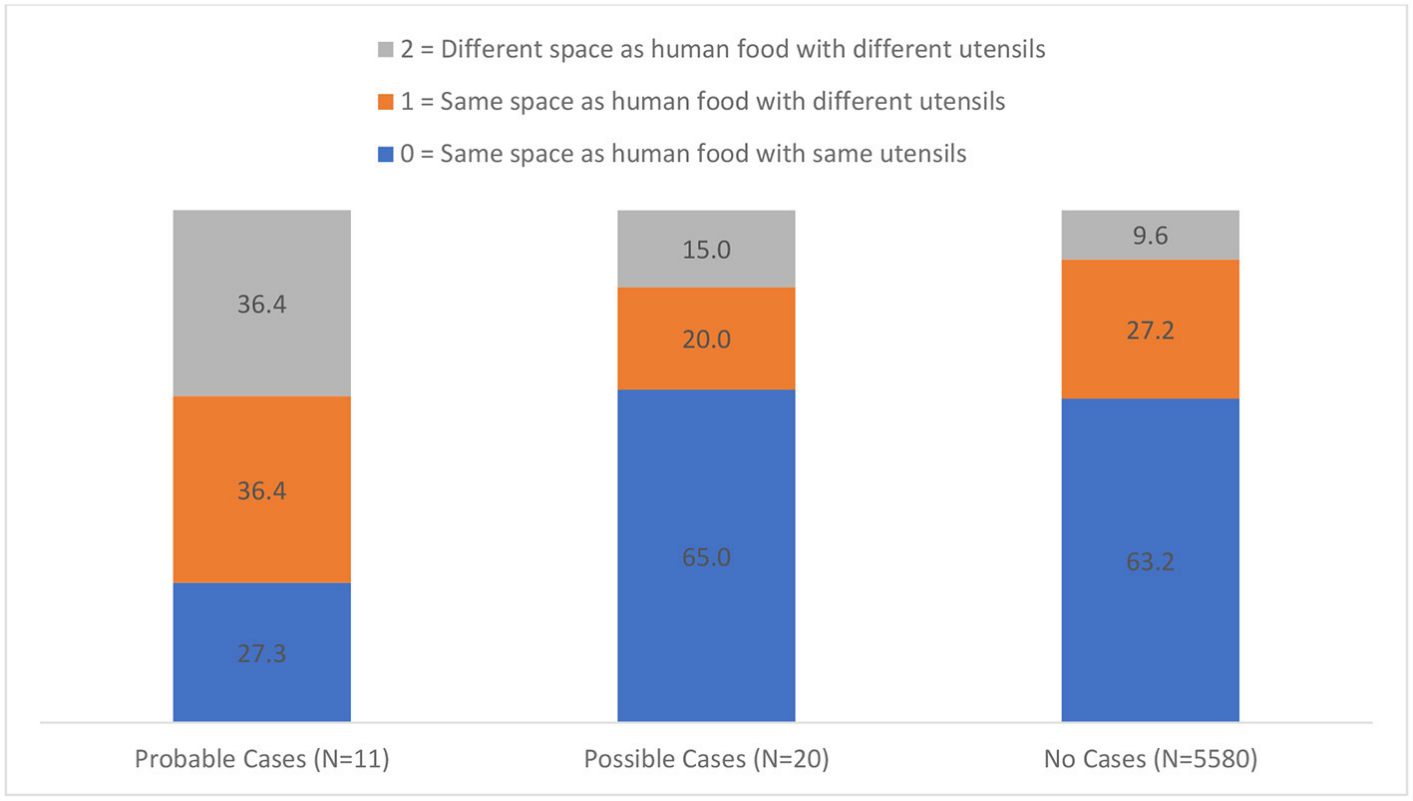
Distribution of probable, possible and no cases of pathogens in households based on where diet was prepared and with what utensils.

Of those surveyed, 63.2% handled MP diets in same place with same utensils as human food, 27.1% handled MP diets in the same place with different utensils as human food and 9.7% handled MP diets in a different place with different utensils as human food. Increased prevalence of pathogens was reported for those HH who prepared the MP diets with different utensils than human food and in a different space than human food (Figure 2). There was no significance between “probable” and “possible” reported cases between MPCD and MPHD, regardless of where and how MP diets were prepared (Table 3).

**Table 3:** Results of 2 sets of univariable logistic regression showing the potential independent predictor variables for the 11 “probable” cases of household transmission with 5,600 controls as well as a total of 31 cases (11 “probable” cases plus 20 “possible” cases) of household transmission with 5,580 controls with numbers and *p*-values reported as with numbers and *P*-values (*P*) for categorical variables and as median values with interquartile range (IQR) and minimum (min) and maximum (max) values and *P*-values (*P*) for continuous variables.

		**11 “Probable” cases**			**31 “Possible” cases**		
**Variable**	**Total**	**Cases**	**Controls**	***P***	**Cases**	**Controls**	***P***
**Demographics**							
**Country of respondent**				1.0			0.2
USA	2,543	6	2,537		16	2,527	
UK	1,858	4	1,854		5	1,853	
Canada	629	0	629		4	625	
Australia	200	0	200		1	199	
Other	381	1	380		5	376	
**Type of pet in HH**				0.8			1
Dog	4,327	10	4,317		24	4,303	
Cat and Dog	929	1	928		5	924	
Cat	355	0	355		2	353	
**Any children in HH**				0.8			0.4
No	3,915	8	3,907		27	3,888	
Yes	1,696	3	1,693		4	1,692	
**Children** ** <2 years**				0.20			0.25
No	5,196	9	5,187		27	5,169	
Yes	415	2	413		4	411	
**Children aged 2–6 years**				0.8			0.8
No	4,997	10	4,987		28	4,969	
Yes	614	1	613		3	611	
**Children aged 7–18 years**				0.6			0.3
No	4,246	9	4,237		26	4,220	
Yes	1,365	2	1,363		5	1,360	
**Adults aged 18–65 years**				1.0			1.0
No	230	0	230		0	230	
Yes	5,381	11	5,370		31	5,350	
**Adults aged** **>65 years**				0.8			0.9
No	4,758	9	4,749		26	4,732	
Yes	853	2	851		5	848	
**Immunocompromised**				1.0			0.13
No	4,651	11	4,640		29	4,622	
Yes	960	0	960		2	958	
**Child in daycare**				0.7			0.4
No	5,258	10	5,248		28	5,230	
Yes	353	1	352		3	350	
*Type of protein sources fed:*							
**Beef**				0.24			0.3
No	458	2	456		4	454	
Yes	5,153	9	5,144		27	5,126	
**Chicken**				1.0			0.8
No	829	0	829		5	824	
Yes	4,782	11	4,771		26	4,756	
**Pork**				**0.03**			**0.02**
No	2,068	8	2,060		18	2,050	
Yes	3,543	3	3,540		13	3,530	
**Lamb**				0.23			0.1
No	1,620	5	1,615		13	1,607	
Yes	3,991	6	3,985		18	3,973	
**Goat**				1.0			0.06
No	4,008	11	3,997		27	3,981	
Yes	1,603	0	1,603		4	1,599	
**Turkey**				**0.001**			**0.001**
No	956	7	949		13	943	
Yes	4,655	4	4,651		18	4,637	
**Duck**				**0.002**			** <0.001**
No	1,619	10	1,609		20	1,599	
Yes	3,992	1	3,991		11	3,981	
**Reindeer**				1.0			0.09
No	5,502	11	5,491		29	5,473	
Yes	109	0	109		2	107	
**Moose**				1.0			0.9
No	5,452	11	5,441		30	5,422	
Yes	159	0	159		1	158	
**Venison/Deer**				0.6			0.9
No	2,619	6	2,613		15	2,604	
Yes	2,992	5	2,987		16	2,976	
**Horse**				1.0			0.8
No	5,195	11	5,184		29	5,166	
Yes	416	0	416		2	414	
**Bison**				1.0			0.5
No	4,607	11	4,596		27	4,580	
Yes	1,004	0	1,004		4	1,000	
**Egg**				0.06			0.1
No	1,187	5	1,182		10	1,177	
Yes	4,424	6	4,418		21	4,403	
**Salmon**				0.4			**0.04**
No	2,367	6	2,361		19	2,348	
Yes	3,244	5	3,239		12	3,232	
**Cod**				0.9			0.6
No	4,508	9	4,499		26	4,482	
Yes	1,103	2	1,101		5	1,098	
**Herring**				0.8			0.8
No	4,416	9	4,407		26	4,390	
Yes	1,195	2	1,193		5	1,190	
**Rabbit**				0.06			**0.02**
No	2,403	8	2,395		20	2,383	
Yes	3,208	3	3,205		11	3,197	
**Other_Fish**				**0.02**			**0.048**
No	2,604	10	2,594		20	2,584	
Yes	3,007	1	3,006		11	2,996	
**Number of protein sources fed**							
**Categorical variable**				**0.03**			**0.04**
1 to 7 sources	2,030	9	2,021		18	2,012	
8 to 10	1,882	1	1,881	0.04	9	1,873	0.04
11 to 18	1,699	1	1,698	0.06	4	1,695	0.06
		**Median**	**IQR**		**Min**	**Max**	
**Continuous variable**		9	5	0.001	1	18	0.001
**Country of origin of raw food**				1.0			0.4
USA	1,700	5	1,695		13	1,687	
UK	1,295	3	1,292		4	1,291	
Canada	422	0	422		4	418	
Australia	181	0	181		1	180	
Canada and/or USA	111	0	111		0	111	
Multiple countries	768	0	768		1	767	
Other	225	0	225		3	222	
Missing	909	3	906		5	904	
**Food sourced from**				0.6			0.7
Multiple Sources	2,970	5	2,965		15	2,955	
Pet Food Shop	1,373	3	1,370		8	1,365	
Online/Internet sales	626	1	625		2	624	
Supermarket/Wholesale	545	1	544		5	540	
Farm, Hunting or Fishing	97	1	96		1	96	
**Pet Food Shop**				0.3			0.09
No	2,238	6	2,232		17	2,221	
Yes	3,373	5	3,368		14	3,359	
**Supermarket/Wholesale**				0.8			0.3
No	2,854	6	2,848		13	2,841	
Yes	2,757	5	2,752		18	2,739	
**Online/Internet sales**				0.4			0.12
No	3,383	8	3,375		23	3,360	
Yes	2,228	3	2,225		8	2,220	
**Farm or Hunter**				0.5			1.0
No	4,133	9	4,124		23	4,110	
Yes	1,478	2	1,476		8	1,470	
**Hunted/Fished**				0.9			0.8
No	5,172	10	5,162		29	5,143	
Yes	439	1	438		2	437	
**Farm/Hunted/Fished**				0.9			1.0
No	3,986	8	3,978		22	3,964	
Yes	1,625	3	1,622		9	1,616	
**Pack size purchased**				0.9			0.8
>1 kg	2,058	3	2,055		16	2,042	
500 g−1 kg	1,058	4	1,054		6	1,052	
<500 g	553	0	553		1	552	
All 3 sizes	819	0	819		3	816	
500 g−1 kg + >1 kg	585	2	583		3	582	
<500 g + 500 g - 1 kg	435	2	433		2	433	
<500 g + >1 kg	103	0	103		0	103	
** <500 g packs**				0.3			0.09
No	3,701	9	3,692		25	3,676	
Yes	1,910	2	1,908		6	1,904	
**500 g−1 kg packs**				0.20			0.5
No	2,714	3	2,711		17	2,697	
Yes	2,897	8	2,889		14	2,883	
**>1 kg packs**				0.22			0.4
No	2,046	6	2,040		9	2,037	
Yes	3,565	5	3,560		22	3,543	
*Storage of raw food once out of freezer*		**Median**	**IQR**		**Min**	**Max**	
**In refrigerator (days)**	5,428	3	2	0.5	0	22	0.5
**At room temperature (days)**	4,449	0.13	0.33	0.7	0	22	0.8
**Ever refreeze**				0.21			0.6
No	3,001	8	2,993		18	2,983	
Yes	2,610	3	2,607		13	2,597	
**Percent raw fed**				0.08			**0.006**
<20% raw	184	2	182		5	179	
20–49%	247	0	247		0	247	
50–89% raw	1,261	4	1,257		8	1,253	
90–100% raw	3,919	5	3,914		18	3,901	
**Percent raw fed**				0.21			0.18
<50% raw	431	2	429		5	426	
50–89% raw	1,261	4	1,257		8	1,253	
90–100% raw	3,919	5	3,914		18	3,901	
**90–100% raw**				0.09			0.16
No	1,692	6	1,686		13	1,679	
Yes	3,919	5	3,914		18	3,901	
**50–89% raw**				0.3			0.7
No	4,350	7	4,343		23	4,327	
Yes	1,261	4	1,257		8	1,253	
**20–49% raw**				NE			NE
No	5,364	11	0		31	5,580	
Yes	247	0	0		0	0	
** <20% raw**				**0.02**			** <0.001**
No	5,427	9	5,418		26	5,401	
Yes	184	2	182		5	179	
**Other_Food_Fed**				0.6			**0.04**
No=Just fed raw	2,518	4	2,514		8	2,510	
Yes=if anything else mentioned	3,093	7	3,086		23	3,070	
**Kibble**				**0.002**			**0.01**
No	4,755	5	4,750		21	4,734	
Yes	856	6	850		10	846	
**Canned**				1.0			0.6
No	5,104	10	5,094		29	5,075	
Yes	507	1	506		2	505	
**Freeze dried**				1.0			0.23
No	4,378	11	4,367		27	4,351	
Yes	1,233	0	1,233		4	1,229	
**Cooked**				0.6			0.8
No	4,815	10	4,805		27	4,788	
Yes	796	1	795		4	792	
**DIY raw**				0.8			0.26
No	2,742	5	2,737		12	2,730	
Yes	2,869	6	2,863		19	2,850	
**Source of food**				0.8			0.22
MP commercial only fed	2,742	5	2,737		12	2,730	
MP home-made only fed	1,116	3	1,113		10	1,106	
MP both types fed	1,753	3	1,750		9	1,744	
*How long has raw food been fed:*		**Median**	**IQR**		**Min**	**Max**	
**Continuously in household**		~3.5	5.33	0.24	0	>12	0.5
**To at least one pet in household**		~4	5.5	0.25	0	>12	0.3
**How raw food handled**				**0.02**			0.06
Same space and instruments	3,545	3	3,542		16	3,529	
Same space, different instruments	1,523	4	1,519		8	1,515	
Different space and instruments	543	4	539		7	536	
**Same space and instruments**				**0.03**			0.19
No	2,066	8	2,058		15	2,051	
Yes	3,545	3	3,542		16	3,529	
**Same space, different instruments**				0.5			0.9
No	4,088	7	4,081		23	4,065	
Yes	1,523	4	1,519		8	1,515	
**Different space and instruments**				**0.008**			**0.02**
No	5,068	7	5,061		24	5,044	
Yes	543	4	539		7	536	
**Does pet(s) also eat**
**Other items**				0.08			0.1
No	2,086	7	2,079		16	2,070	
Yes	3,525	4	3,521		15	3,510	
**Feces**				0.4			0.8
No	4,617	8	4,609		25	4,592	
Yes	994	3	991		6	988	
**Soil/Grass**				0.1			0.4
No	3,148	9	3,139		20	3,128	
Yes	2,463	2	2,461		11	2,452	
**Dead animals from the forest**				0.3			0.5
No	5,106	9	5,097		27	5,079	
Yes	505	2	503		4	501	
**Spoiled food**				1.0			0.8
No	5,319	11	5,308		29	5,290	
Yes	292	0	292		2	290	
**Garbage**				0.22			0.9
No	5,460	10	5,450		30	5,430	
Yes	151	1	150		1	150	
**Pigs ears**				0.4			0.7
No	4,664	8	4,656		25	4,639	
Yes	947	3	944		6	941	
**Drink from puddles/other standing water**				0.4			0.7
No	3,424	8	3,416		20	3,404	
Yes	2,187	3	2,184		11	2,176	
**None of the above**				0.09			0.1
No	3,509	4	3,505		15	3,494	
Yes	2,102	7	2,095		16	2,086	

*Bolded values are statistical significance*.

### Household Non-MP Diet Pathogen Considerations

Respondents indicated their pets consuming other animal feces, soil/grass, dead animals, spoiled food, garbage, pig ears, and standing water at 17.7, 43.9, 9, 5.2, 2.7, 16.9, and 39.9%, respectively. Of those HH with cats (*n* = 929), 48.4% indicated their cat was outside always or sometimes. Our data did not show any significant risk from any one of these risk factors (Table 3).

### Potential Transmission of Foodborne Pathogens

Eleven HH (0.20%; 95% CI, 0.10–0.36) were classified as having experienced “probable” transmission, and an additional 20 HH (0.36%; 95% CI, 0.22–0.56) were classified as having experienced “possible” transmission to result in a total of 31 HH (0.55%; 95% CI, 0.38–0.79) being identified as being potential cases of transmission (Tables 1, **6**). Our data or analysis did not indicate that the type of MP diet (MPCD or MPHD) was a significant risk factor for any “probable” or “possible” reported cases (Table 3). The remainder of HH (*N* = 5,580 = 99.45%; 95% CI, 99.21–99.62) were not considered to have experienced potential transmission of foodborne pathogens based on their responses in the survey.

All of the respondents from the 11 “probable” HH were also able to name a pathogen in response to the question: “#32—Which pathogens(s) was verified in the fecal sample by the laboratory? (check ALL that apply)” (responses included either *Campylobacter* or *Salmonella* or *Salmonella* and *Escherichia coli*); eight of them also responded Yes to the question: #33—Was this pathogen the SAME pathogen that was found in the raw meat pet food? Four also responded Yes to the question: #38—Did the family pet(s) have any symptoms at the same time when people got sick?

The most frequently reported pathogens were *Salmonella* (*n* = 11, 0.2%), *Campylobacter* (*n* = 6, 0.1%), *E. coli* (*n* = 4, 0.1%), with the most common age group being adults age 18–65 (*n* = 29, 78.4% of cases). The next most common age group impacted was children under 2 years of age (*n* = 3, 8.1% of cases) followed by two cases each reported in both 2- to 6-year-old age group and in the over 65 age group representing 0.6% of overall reported cases each.

### Logistic Regression Analysis

The results of univariable analysis are reported for 72 variables with some overlap/duplication of variables due to different methods of coding for statistical analysis (Table 3). Beef, chicken, and egg were the most commonly reported proteins that HH said they fed, although none of these protein sources were significant variables in univariable analysis, and they were not retained in the final models. Feeding of pork, turkey, duck, rabbit, salmon, and other fish showed significance in univariable analysis, suggesting that feeding of these proteins was associated with a lower risk of reported transmission, although none of these variables were retained in the final models. Instead, it was the total number of proteins fed that was a significant predictor of both “probable” and “possible” cases; responses indicated that 82% of “probable” cases and 58% of “possible” cases were feeding 7 or less MP proteins, 9% and 29% were feeding 8–10 MP proteins, and only 9 and 13% were feeding 11–18 MP proteins for the “probable” and “possible” cases, respectively (Tables 3–5).

**Table 4:** Results of multiple logistic regression for the 11 “probable” cases of household transmission and 5,600 controls showing the potential independent predictor variables in the final model with numbers of cases and controls with *p*-values, odds ratios (ORs) and 95% confidence intervals (CIs).

					**Univariable analysis**			**Multivariable analysis**		
**Variable**	**Category**	**Total**	**Cases**	**Controls**	**OR**	**95% CI**	***P***	**OR**	**95% CI**	***P***
**Kibble**						**0.002**			**0.008**
	No	4,755	5	4,750	Ref^*^			Ref		
	Yes	856	6	850	**6.7**	**2.0–22.0**		**5.2**	**1.5–17.5**	
**Number of different raw protein sources fed**					**0.72**	**0.58–0.88**	**0.001**	**0.75**	**0.62–0.92**	**0.006**
**How raw food handled**				**0.02**			**0.02**			
	Same space and instruments	3,545	3	3,542	Ref			Ref		
	Same space, different instruments	1,523	4	1,519	3.1	0.7–13.9	0.14	3.2	0.7–14.4	0.13
	Different space and instruments	543	4	539	**8.8**	**2.0**–**39.3**	**0.005**	**8.3**	**1.8**–**37.7**	**0.006**
Constant								**0.029**		** <0.0001**

* *Ref = Referent category*.

*Nagelkerke R Square = 0.165*.

*Hosmer and Lemeshow goodness-of-fit Chi-square test: χ^2^ = 6.179 with 8 degrees of freedom, P = 0.63. Bolded values are statistical significance*.

**Table 5:** Results of multiple logistic regression for a total of 31 cases (11 “probable” cases plus 20 “possible” cases) of household transmission and 5,580 controls showing the potential independent predictor variables in the final model with numbers of cases and controls with *p*-values, odds ratios (ORs) and 95% confidence intervals (CIs).

					**Univariable analysis**			**Multivariable analysis**		
**Variable**	**Category**	**Total**	**Cases**	**Controls**	**OR**	**95% CI**	***P***	**OR**	**95% CI**	***P***
** <20% Raw fed**						**0.0004**			**0.002**
	No	5,427	26	5,401	Ref^*^			Ref		
	Yes	184	5	179	**5.8**	**2.2–15.3**		**4.6**	**1.7–12.5**	
**Number of different raw protein sources fed**					**0.72**	**0.58–0.88**	**0.001**	**0.85**	**0.76–0.95**	**0.004**
**How raw food handled**							**0.02**			**0.02**
	Same space and same/different instruments	5,068	24	5,044	Ref			Ref		
	Different space and instruments	543	7	536	**2.8**	**1.2–6.4**		**2.7**	**1.2–6.4**	
Constant								**0.015**		** <0.0001**

* *Ref = Referent category*.

*Nagelkerke R Square = 0.059*.

*Hosmer and Lemeshow goodness-of-fit Chi-square test: χ^2^ = 11.408 with 8 degrees of freedom, P = 0.18. Bolded values are statistical significance*.

The final model for the 11 “probable” cases of household transmission included three variables that explained 16.5% of the variability in the data: when kibble was fed in addition to raw, fewer different raw protein sources were fed, and if the raw food was handled using different place and utensils compared to using the same place and utensils were all associated with a higher risk of HH transmission (Table 4). The final model for the 31 “possible” cases of HH transmission included three variables, which explained 6% of the variability in the data: when <20% of the diet fed was raw, fewer different raw protein sources were fed, and if the raw food was handled using different space and instruments compared to using the same space and the same or different instruments were all associated with a higher risk of HH transmission (Table 5).

While the country of respondent and the country of origin of raw food were not significant and were not put forward for inclusion in the final models, when one or both of these variables was included as a fixed effect in a sensitivity analysis, the results were similar to the models that did not control for either of these variables. Similarly, adding into the final models either the two-category variable for MPCD or the three-category variable for MPHD only, MPCD only or a combination of the two types of MP diets did not change the models and neither of these variables were significant.

## Discussion

This study had similar results to that of Anturaniemi et al. (18), demonstrating that MP diets rarely resulted in reporting of pathogen transmission to human members of HH (Table 6) and that there were a number of known and unknown factors also at play. Our results showed a similarly low reported prevalence of suspected pathogen transmission, leaving an overwhelming large proportion (>99%) of HH in both studies feeding raw food to their pets and reporting that they were not affected by potential transmission of foodborne pathogens. For our study, none of the household-reported cases submitted laboratory reports to confirm their claim even though respondents were asked to do so. This may indicate that testing for pathogen-related illness is not first line in veterinary medicine or human medicine, which could mean that reports are being suspected and treated without confirmation. The lack of confirmed laboratory reports could also mean that potential pathogen-related illness could be going undetected altogether.

**Table 6:** Comparison of the current study with the DogRisk study.

	**Reported cases of transmission**									
	**Confirmed/Probable**		**Suspected/Possible**		**Total**		**No transmission**		**Total**	
**Study**	**%**	**(95% CI)**	**%**	**(95% CI)**	**%**	**(95% CI)**	**%**	**(95% CI)**	***N***	**%**
DogRisk	0.24	(0.17–0.33%)	0.15	(0.10–0.22%)	0.38	(0.30–0.49)	99.62	(99.51–99.70)	16,475	100
Current	0.20	(0.10–0.36)	0.36	(0.22–0.56)	0.55	(0.38–0.79)	99.45	(99.21–99.62)	5,611	100

In the case of gastrointestinal-related symptoms in humans, or pets, it is our observation that laboratory testing to determine presence of pathogens is not a standard first-line practice, which complicates identification of pathogen cases related to all diet types. Complicating matters, it is also possible for seemingly healthy dog or cat guts to contain such pathogens without any sign of illness, regardless of diet. *Salmonella* and *E. coli* are also known to be shed in the feces of cats and dogs regardless of diet, yet risk to humans has not been established. The presence of pathogens in the diet may not translate to shedding of said pathogens in feces, as one study demonstrated that despite *Salmonella* detection in 80% of chicken MP diets, only 30% of canines shed *Salmonella* in their feces (19).

We do note the highest number of cases in our study were reported with beef and chicken proteins, followed by egg. Care should be taken to ensure that this is not taken out of context, since beef and chicken proteins are the most common proteins regardless of pet food format. However, we note that none of the proteins within this study are associated with increased risk of illness in humans. On the other hand, the CDC does recognize that these and other proteins are often contaminated with pathogens (20, 21), which indicates the importance of proper education regarding food safety and handling practices.

We did find that those HH who feed more protein sources appear to have a lesser incidence of possible or probable pathogen cases than HH who fed less variety. Feeding 11 or more different proteins showed a lesser prevalence of possible or probable cases of pathogen transmission to humans, while feeding seven or less protein sources did appear to increase risk. In our data, 18 out of 31 possible and probable cases (58%) were feeding 7 or less proteins, and 87% of cases were feeding 10 or less proteins. Only four “possible” or “probable” cases were reported in those feeding 11 or more proteins. These data indicate that there may be some potential for lesser risk of pathogen transmission with a varied diet potentially because more advanced feeders tend to feed a greater variety while also having more effective handling and hygiene routines. However, more research is needed to determine what factors are influencing this potential outcome whether it be related to nutrients, positive modulation of the gut microbiota, health status of the animal, sourcing of the diet, and/or cleaning and handling practices within HH.

Mixing food forms such as feeding kibble with raw was associated with an increased risk of probable and possible cases of pathogen transmission (Figure 1), which was also similar to Anturaniemi et al. (18). Our data show that feeding 20% or less MP diets translated to an increased risk, while feeding 90% or more MP diets demonstrated a decreased risk. This could be due to contamination of other diet forms, product being compromised during distribution or storage, and different handling practices among the two forms or other known or unknown factors. It is likely that more advanced and experienced MP diet feeders are not using kibble and are likely utilizing more effective hygiene and handling techniques. More research is needed to determine if mixing diet forms is a true risk factor. Previous research has shown that owners who feed low moisture foods (e.g., dry foods, treats) do not perceive such products as having pathogenic risk associated with them despite numerous documented cases of pathogens in dry pet food products and treats (11, 12, 22–25). Furthermore, the presumption bias of owners assuming dry pet foods and treats were of minimal or no risk resulted in only 58% of pet owners washing their hands after feeding their pets (25). Considering this, it is logical that cross-contamination from dry pet food and treats is a risk factor associated with pathogens regardless of the presence of MP diets in the household.

The type of MP diet is important when considering risk of pathogenic contamination of the diet and thus risk of pathogenic infection of the pet or humans in the household. As stated, risk of pathogens in MPCD in the USA have the same zero-tolerance policy for pathogens as other commercial pet food formats (e.g., kibble). For context, a recent study highlighted various pathogens (*E. coli, Campylobacter*, and *Salmonella*) detected in 18 of 25 fecal samples of dogs fed MP diets. Comparatively, pathogens were detected in 5 of 25 fecal samples of dogs fed dry diets (26). The authors did not differentiate whether dogs in the MP diet fed group were fed MPCD or MPHD, which is critical for determining risk factors in each country. If fed MPHD diets, this finding makes sense because we know that the human food supply in many countries harbor many of these pathogens, as they are sold with the intent of being cooked. It is also clear that many in this group also mixed diet forms (e.g., MP and kibble), which, per our data, increases risk for pathogenic transmission. We are also unaware if HHs in this study were practicing proper food safety (e.g., food storage, cleaning bowls), what other environmental exposure factors the pet was exposed to, and what types of proteins sources were being fed. Additionally, if MPHD were used, sourcing from grocery or hunting wild animals would provide additional risk factors not likely considered.

Another factor to consider is that many pet owners are not aware of proper preparation and cleaning when feeding MP diets, or pet food in general, especially in the USA. For example, many kibble-feeding HHs do not often clean and sanitize pet food bowls daily, which have been found to be one of most likely items in the household to be contaminated with pathogens (27). It could be hypothesized that MP-diet HH would be more vigilant in cleaning food bowls; however, the authors are not aware of any data to confirm this. Considering this, it is likely that there is a disconnect in pathogen risk factors between different feeding formats (e.g., kibble and MP diets), meaning that MP-diet-feeding HH likely have more effective handling and hygiene routines since kibble and heat-treated diets are perceived as safe. Regardless, it is likely that pet owners are in need of more robust food safety education no matter what pet food format is fed.

In our study, how and where MP diets were prepared also appears to influence risk of pathogen transmission. These results are in line with data reported by Anturaniemi et al. (18), in that preparing MP diets in the same place as human food with the same utensils may provide a protective effect against pathogens. MP diets that were both prepared in the same place but with different utensils and in a different place with different utensils had higher reports of pathogen transmission per our data. This could be a result of owners cleaning their own utensils and surfaces more diligently than ones solely used for pet food and further underscores the importance of proper food safety and hygiene techniques.

Proper food safety manufacturing and handling protocols are equally as important as pet owners handling said pet food, including MP diets, because people handling the food and the environment in which the food is handled could also be the source of contaminants, not just the food itself. For example, the European Pet Food Industry recognizes risk associated with MP diets and has proactive pet food safety campaigns that aim to educate the public on proper MP diet food safety initiatives such as food handling, storage, and handwashing (28). Such initiatives are lacking in the USA, and instead, the public is discouraged in feeding MP diets.

Considerations for other risk factors associated with pathogen transmission would be various human and pet interactions independent of diet format. Those interactions would include but are not limited to letting the pet lick family members' faces, letting the pet on furniture, hand hygiene, and household cleaning practices. Thomas and Feng (25) found that over 50% of pet owners shared their bed with pets and that the majority of pet owners allowed pets to lick human faces and have close prolonged contact. HHs with canines are far more likely to feed treats and leftover human food products to their dogs (25), and thus, these activities also present pathogen risks into the household independent of MP diets, albeit the quantifying risk of pathogen transmission via such activities has never been established. Similar to MP diets, it would be difficult, if not impossible, to isolate each of these variables to determine true risk.

### Minimal Cases of Pathogens Reported

Low reporting of suspected transmission of pathogens overall may be due to factors such as owner and/or veterinarian assumption of pathogens or illness due to confounding variables including animal eating garbage, consumption of pig ear treats, drinking standing water, cats having free access to the outside unsupervised, and/or young children in daycare, among other known and unknown variables. Additionally, a concern among MP diet feeders often echoed on social media channels is the hesitation or failure to disclose to their veterinarian that they are feeding MP diets to their pet due to fear of receiving pressure to switch their pet to commercial or veterinary diets. Concerns have also been raised in veterinary communities that veterinary resistance to MP diets may result in pet owners to not disclose feeding of MP diets, which could risk damaging client–veterinarian relationships (29) and potentially reduce frequency of routine care or lack of care altogether. In the context of pathogens, if a veterinarian is not aware of a cat or dog eating MP diets, this would prevent MP diets from being considered a factor in potential or confirmed cases of pathogen infection. Another reason for the hesitation or failure of owners to disclose MP diets to their veterinarian may be due to some veterinarians refusing to see pets that consume MP diets, at least in the USA. These actions by members of the veterinary community are likely negatively impacting pet care and could result in lack of reported MP-diet pathogen cases, identification of nutrient concerns, and other health concerns independent of diet.

Increased reporting of contamination and recalls of MP diets in the marketplace in several countries may be leading to “assumed” pathogen infection secondary to MP diets without laboratory confirmation by the veterinarian. This is problematic, as it may fail to identify or delay identification of an outbreak from MP diets or other sources, which in either case leaves the public at risk (i.e., raw materials, environmental contamination, etc.). Additionally, assumption bias in these cases is likely leading to overuse or misuse of antibiotics, which is another significant concern for human and animal health.

In the USA, the FDA and state agriculture departments proactively and routinely test products in the marketplace for pathogens and contaminants. A positive result for any pathogen or contaminant in any food format results in an automatic recall whether voluntary or involuntary because the food is considered “adulterated.” However, recalled products do not always cause animal or consumer illnesses or deaths. Challenges in determining source of pathogenic transmission in animals or humans, or current recall status of products, should not be used to assume the presence of pathogens especially in the case of ill pets, ill humans, or pet food. Confirming the presence of pathogen infection in the pet and/or pet food that is suspect of involvement of pathogen illness or contamination is still necessary. We suggest that the human medicine, veterinary medicine, and pet food manufacturing communities devise a standardized protocol for the consistent investigation of suspected incidents of pathogen contamination for humans, pets, pet food, and/or other variables regardless of suspected medium.

### Pet Food Policy and Pathogens

The same pathogens that are warned against MP diets have been present and have caused issues in cooked products including cooked foods, dry kibble, and pig ear treats. In recent history, the US Food & Drug Administration (FDA) has posted multiple warning letters and recalls, including voluntary recalls, for such product (11, 23, 24, 30, 31). The FDA and US veterinary organizations largely discourage the use of MP diets, while European organizations like European Pet Food Industry (FEDIAF) provide MP diet handling and food safety information to the public to help mitigate risk by educating the public (28). Despite different views and increased reports of pathogen contamination or detection, including voluntary recalls and advisories of MP diet products, little attention has been given to investigating pathogen mitigation steps, feeding practices of pet owners, and actual prevalence of pathogen transmission in MP diet HH.

Considering the Food Safety Modernization Act (FSMA) and the FDA's zero-tolerance policy, MP-diet manufacturers should be proactively identifying risks such as sources of contamination, handling practices, and even risk associated with protein sources. Such practices would be benefical, as they would allow mitigation measures to be developed and implemented to reduce such risks. When it comes to pet food, the USA takes a “zero-tolerance” approach, meaning that any pet food with pathogens present is considered adulterated regardless of format. It is also important to note that pathogen risks are likely to differ from country to country or even state to state depending on agricultural, slaughter, and other food safety practices. Anti-MP-diet groups, such as the AVMA, should recognize that many consumers are feeding MP diets and embrace dialogue and investigate methods to ensure nutritional adequacy and safety and support research in determining best ways to mitigate risks of all diets. MP diet popularity is increasing year after year, and identifying companies who are accountable to third party safety and nutritional data in addition to supporting companion animal nutrition research is likely to help mitigate risk of pathogens among MP diets.

FEDIAF in Europe, which is similar to AAFCO in the USA, has taken a different approach to pathogens in pet food. FEDIAF recognizes that pathogens are present in the food supply and therefore likely to be present within MP diets. Additionally, they recognize that pet owners are likely to continue feeding MP diets despite discouragement of such practices from any regulatory or veterinary authority. Instead, FEDIAF has been proactive with campaigns aimed to educate pet owners on responsible and proper food safety and handling techniques to mitigate risk. In addition to efforts by FEDIAF, European veterinarians and manufacturers have made several steps to improve the safety, sustainability, and nutrition of MP diets. The Pet Food Manufacturers Association (PFMA) has launched an initiative promoting best practices for MP diets within Europe by improving food safety, defining specific MP diet regulatory requirements and more.

The FDA, AVMA, AAHA, and others warn Americans against feeding MP diets to their pets, despite the fact that MPCD are held to the same policies (e.g., zero tolerance for pathogens) as kibble and canned diets. This could be the result of lack of awareness and education of all the standards that are required for all dog and cat foods. Although many may believe that minimally processed diets may pose a greater risk for microbial contamination, this risk does not go away with highly processed (HP) diets as previously discussed. The industry would benefit greatly from educating consumers on food safety and handling practices regardless of the diets they feed. The designation between MPCD and MPHD are lacking within FDA, AVMA, and AAHA policies, and these policies would benefit from an update that designates between the two. By further defining the MP category would differentiate between potential microbial risks, since human grade raw ingredients (i.e., grocery foods) used to make MPHD have permissible levels of microbes while MPCD do not.

## Limitations

This study has many limitations. Owner-reported data are often unreliable and/or incomplete. This was evident in that some respondents could not name a pathogen for their potential or probable case. In addition, we received zero laboratory confirmed cases, which questions the validity of reported probable and possible cases. In addition, there is considerable presumption bias within pro- and anti-MP diet communities. Often, these sentiments are fueled by incomplete or inaccurate information on social media and other various outlets. When conducting this survey, the authors were met with much hesitation and often abrasive responses from the pro-MP diet community assuming that the authors were anti-MP diet. Examples of such included large social media platforms and influencers advising MP diet feeders to not participate in the survey. This is significant, since many pro-MP diet feeders have indicated they obtain the majority of their information from television news and social media. This likely led to decreased survey participation from the MP diet feeding community, particularly in the USA. Comparatively, resistance was met within some members of the veterinary community when distributing this survey. Additionally, lack of first-line testing for foodborne illness regardless of diet due to personal and or veterinary perception of safety of any diet form is a confounder. Owner finances also have a similar impact, as the cost of testing is a barrier to testing each and every expected case. There is potential bias among current raw feeders that MP diets are inherently safe due to lack of or underreported cases. It is likely that confirmed cases are underreported or not reported at all for one or all of these reasons.

While this study was designed specifically for those who feed MP diets, the need for a similar study with other food formats for comparison is apparent; however, the authors also recognize that conducting such a study would be difficult, since other food formats such as kibble and canned diets are perceived as safe. The perceived safety, for reasons discussed above, create a bias when pets and people present with symptoms that could relate to pathogenic illness, which means that cases would likely not be recognized timely, or at all. This is supported by the fact that most cases related to pathogenic illnesses from MP diets are also not being confirmed by laboratory testing and rather assumed to be pathogenic in nature.

The COVID-19 pandemic also presented challenges, as a limited number of responses were collected due to the survey being opened a lesser time than expected. It is also possible that some GI symptoms for “possible” cases could have been associated with GI symptoms of COVID-19.

## Conclusion

Our study found a low risk of transmission of pathogens relating to MP diets, although it was clear that there was a significant lack of testing to confirm or rule out the potential for pathogen infection whether or not MP diets were associated with potential cases. Both the veterinary and human medical communities are in need of clear directives and protocols for identifying potential pathogen cases in relation to public and animal health regardless of diet type. Additionally, the terminology to define the type of MP diet is necessary, as MPCD and MPHD do carry different risk factors, and future work assessing MP diets for pathogens, nutritional adequacy, or otherwise should differentiate between MPCD and MPHD in order to more accurately identify risk factors and areas of concern and improvement. Based on the results of this survey, confirmed pathogen transmission from MPCD and MPHD diets to humans appears to be rare. We conclude that potential or probable cases of pathogen transmission is likely dependent upon hygiene and food safety measures, and more education surrounding food safety should reduce risk.

## Data Availability Statement

The raw data supporting the conclusions of this article will be made available by the authors, without undue reservation.

## Author Contributions

NC and RY were responsible for study design and data collection. VA analyzed the data. NC wrote the manuscript. RY and VA edited the manuscript. All authors contributed to the article and approved the submitted version.

## Conflict of Interest

NC owns and operates Northpoint Pets and Company. RY is co-founder of Guardian Pet Food Company, Advisor for Bond Pet Foods and Senior strategist for KDC AgriBusiness. The remaining author declares that the research was conducted in the absence of any commercial or financial relationships that could be construed as a potential conflict of interest.

## Publisher's Note

All claims expressed in this article are solely those of the authors and do not necessarily represent those of their affiliated organizations, or those of the publisher, the editors and the reviewers. Any product that may be evaluated in this article, or claim that may be made by its manufacturer, is not guaranteed or endorsed by the publisher.
